# Application of Dexmedetomidine Sedation Combined With Suprascapular Nerve Block and Axillary Nerve Block in Shoulder Arthroscopy, A Randomized Single-Blind Study

**DOI:** 10.3389/fphar.2021.740385

**Published:** 2021-12-20

**Authors:** Chu-Ting Wang, Liang-Ming Zhu, Ji-Ling Wu, Fen-Fen Kang, Zhi-Jian Lin

**Affiliations:** ^1^ Department of Anesthesiology, Zhangzhou Affiliated Hospital of Fujian Medical University, Zhangzhou, China; ^2^ Department of Emergency, Zhangzhou Affiliated Hospital of Fujian Medical University, Zhangzhou, China

**Keywords:** dexmedetomidine, shoulder arthroscopy, cerebral blood perfusion, suprascapular nerve block, axillary nerve block

## Abstract

**Objective:** The aim of the present study was to evaluate the anesthetic and analgesic effects of dexmedetomidine combined with suprascapular nerve block and axillary nerve block in shoulder arthroscopy.

**Methods:** A total of 60 patients were randomly divided into the experimental group (DEX group) and the control group (GA group) via a random number table method. Dexmedetomidine sedation combined with suprascapular nerve block and axillary nerve block was used in the DEX group, while general anesthesia with tracheal intubation combined with interscalene brachial plexus block was used in the GA group. The perioperative indexes, intraoperative hemodynamics, cerebral oxygen saturation, and postoperative pain score, as well as any complications, were compared between the two groups.

**Results:** The anesthesia duration (*p* < 0.05) and postoperative monitoring time (*p* < 0.05) in the DEX group were significantly shorter than those in the GA group. At most time points during the anesthesia, the cerebral oxygen saturation (*p* < 0.05) and mean arterial pressure (*p* < 0.05) in the DEX group were significantly higher than those in the GA group. Additionally, the decrease in the cerebral oxygen saturation and mean arterial pressure in the GA group was significantly higher than that in the DEX group (*p* < 0.05). The pain score of DEX group 12 h after operation significantly lower than that in the GA group (*p* < 0.05), and the incidence of postoperative hypoxemia along with nausea and vomiting in the GA group was significantly higher than that in the DEX group (*p* < 0.05).

**Conclusion:** Dexmedetomidine combined with suprascapular nerve block and axillary nerve block could reduce the incidence of hypoxemia, while the approach demonstrated better hemodynamic stability, fully ensured the cerebral blood perfusion, and exhibited better anesthetic and analgesic effects, meaning it could be safely and effectively applied in shoulder arthroscopy procedures.

## 1 Introduction

As the joint with the largest range of motion, the shoulder plays a crucial role in our daily and working lives, and shoulder diseases often have a serious impact on the individual’s quality of life. With the development of modern medical technology, shoulder arthroscopy has been widely used in the examination, diagnosis, and treatment of shoulder joint diseases (Choi et al., 2015). Due to the special requirements of shoulder arthroscopy in terms of the operation position, the patient should be placed in the beach-chair position. The degree of intraoperative cooperation in patients and the postoperative analgesic requirements are relatively high due to the stimulation of the intra-articular nerves and the traction of the muscle. Currently, general anesthesia combined with interscalene brachial plexus block is commonly used (Wei et al., 2012; Li et al., 2013; Li et al., 2020).

However, in recent years, studies both at home and abroad have shown that general anesthesia has a major impact on the respiratory and circulatory system (Koh et al., 2013; Verelst and van Zundert, 2013), which means postoperative respiratory depression or hypotension can easily occur. Specifically, the cerebral blood perfusion will also be affected, and the interscalene brachial plexus block may also lead to paralysis of the phrenic nerve, resulting in postoperative respiratory dysfunction. Meanwhile, the effective sedative effect and good respiratory and circulatory stability of dexmedetomidine, a highly selective α2-adrenoceptor agonist, have been confirmed in clinical practice. In addition, a selective nerve block can provide effective analgesia without affecting the respiratory functioning. The present study aims to evaluate the anesthetic and analgesic effects of dexmedetomidine combined with suprascapular nerve block and axillary nerve block in shoulder arthroscopy in view of exploring a safer and more effective anesthesia method.

## 2 Materials and Methods

### 2.1 The Study Subjects

Sample size estimation: the cerebral oxygen saturation was used as the main observational index in this study. Finally, a total of 60 patients undergoing selective shoulder arthroscopy at our Hospital were recruited from June 2019 to December 2019.

The inclusion criteria were as follows: 1) patients aged between 18 and 60, 2) patients with American Society of Anesthesiologists (ASA) grades I–II, and 3) patients with normal coagulation functioning. Meanwhile, the exclusion criteria were as follows: 1) patients with cerebrovascular diseases, 2) patients with orthostatic hypotension, 3) patients who are allergic to local anesthetics, 4) patients with mental illness complications, 5) patients with a history of drug abuse, and 6) patients with an injured, deformed, or infected puncture site. Finally, the withdrawal criteria were as follows: 1) patients who failed to complete the arthroscopy-based operation and who subsequently underwent incision surgery.

### 2.2 Grouping

Following the approval of the ethics committee of our Hospital (approval no. 2018-lx-005), the study was fully explained to the patients and their families before they signed the informed consent form. A total of 60 patients who satisfied the inclusion criteria will be randomly divided in a 1:1 ratio to the DEX group and GA group. Randomization will be performed on the basis of permuted block sizes of 4. Randomization sequences will be prepared according to concealed random allocation from a computer-generated random numbers table. To ensure blinding, randomization sequences will be kept in opaque, sealed and sequentially numbered envelopes. Only the operators were accessible to know the study grouping.

### 2.3 Anesthesia Method

With the subjects in the operating room, their venous channels were established before routine ECG monitoring and radial artery blood pressure monitoring (Draeger Infinity M540 ECG monitor) were applied. Meanwhile, local non-invasive cerebral oxygen saturation monitoring (FORE-SIGHT MC-2030C brain oxygen saturation monitor) was also performed. The anesthesia methods are outlined below.

#### 2.3.1 The Experimental Group (DEX Group)

This group involved the administration of dexmedetomidine combined with suprascapular nerve block and axillary nerve block. With the patients in the operating room, they were given 3 L/min of mask oxygen inhalation and an intravenous infusion of dexmedetomidine with an initial dose of 0.1 μg/kg/min and a pumping duration of 10 min. The Ramsay scale was used to assess the degree of sedation. When the score was stable at around 3–4, ultrasound-guided suprascapular nerve block combined with the axillary nerve block was administered by one anesthesiologist. For the suprascapular nerve block, the patients were placed in the sitting position and an ultrasound transducer (6–13 MHz) (SonoSite, Seattle, WA, United States) was initially placed parallel to the margo superior of scapular spine. The transducer was then moved down in search of suprascapular notch. Then, the transducer was rotated 30° in order to identify the suprascapular arteries and veins as well as suprascapular nerves. An in-plane ultrasound-guided puncture was performed by a 5 cm nerve block from outside to inside. The tip of the disposable sterile puncture needle (Beijing Chang chuan Co., Ltd., China, Beijing) was positioned around nerve sheath of suprascapular nerves. After identifying the position of the needle tip, 5–10 ml of 0.5% ropivacaine was injected slowly under continuous ultrasound control. Axillary nerve block was performed just after suprascapular nerve block. The ultrasound transducer (8–14 MHz) was placed in a sagittal plane 4 cm inferior to the posterolateral of the acromion, in search of quadrangular space. The transducer was then moved transversely in order to identify posterior circumflex humeral vein and artery accompanied by axillary nerve. An in-plane ultrasound-guided puncture was performed by a 5 cm nerve block needle through the deltoid muscle. The tip of the needle was positioned just above the posterior circumflex humeral artery. After identifying the position of the needle tip, 5–10 ml of 0.5% ropivacaine was injected slowly.

During the course of the anesthesia, dexmedetomidine was infused at a rate of 0.2–0.7 μg/kg/h until the end of the operation. With reference to the heart rate and blood pressure during the operation, the drug dosages were adjusted, with the dosages reduced in the elderly patients and in those exhibiting hepatic and renal dysfunction to ensure that the sedation score remained stable at around 3–4. Following the operation, the patients were transferred to the anesthesia recovery room for continuous monitoring. In the case of an imperfect anesthesia effect, sevoflurane inhalation was administered to assist with the sedation. If this proved ineffective, general anesthesia under endotracheal intubation would have to be used to align with the operation. The anesthesia duration time was measured from the initiation of pumping dexmedetomidine to the recovery of orientation and spontaneous breathing.

#### 2.3.2 The Control Group (GA Group)

This group involved the use of a general anesthesia with tracheal intubation combined with interscalene brachial plexus block. With the patient in the operating room, 0.03 mg/kg of midazolam, 2 mg/kg of propofol, 0.3 μg/kg of sufentanil, and 0.15 mg/kg of cisatracurium were used for anesthesia induction before endotracheal intubation was performed. Then, ultrasound-guided interscalene brachial plexus block was performed using a 0.5% ropivacaine solution (15–20 ml). During the course of the anesthesia, the patients were ventilated with intermittent positive pressure ventilation. Air and oxygen were mixed for ventilation, while the end expiratory concentration of sevoflurane was maintained at between 1 MAC and 1.5 MAC. Remifentanil was continuously pumped at a rate of 0.1–0.15 μg/kg/min until the end of the operation, while cisatracurium was added in accordance with the situation. Here, various vasoactive drugs could be used to assist in the regulation of the blood pressure. Following the completion of the operation, neostigmine and atropine were administered to antagonize the effects of the muscle relaxants. Once the patient was awake and had recovered to spontaneous breathing, the tracheal tube was withdrawn and the patient was transferred to the anesthesia recovery room for continuous monitoring. The anesthesia duration time was measured from the initiation of anesthesia induction to the recovery of orientation and spontaneous breathing.

#### 2.3.3 Patient-Controlled Analgesia

To alleviate the postoperative pain and to improve the level of comfort, all the patients underwent patient-controlled analgesia (PCA) at the end of the operation. Here, 150 μg of sufentanil, 100 mg of flurbiprofen axetil, and 5 mg of tropisetron were diluted to 100 ml with 0.9% normal saline. Meanwhile, the total volume of the analgesia pump was 100 ml, while the continuous infusion rate was 2 ml/h. The patients were allowed to operate the analgesia pump according to their own needs. The single-press dose was 0.5 ml, while the locking time was 15 min and the application duration was 48 h.

### 2.4 Observation Indexes

The main observation indexes were the intraoperative mean arterial pressure, the fluctuation of the cerebral oxygen saturation, the postoperative pain visual analogic scale (VAS), and any emerging complications. Meanwhile, the secondary indexes included the anesthesia duration, the operation duration, and the monitoring time in the anesthesia recovery room (the time from entering the resuscitation room after operation to leaving the resuscitation room).

### 2.5 Statistical Analysis

The collected data were inputted into SPSS software. The continuous variables were summarized as means (standard deviation) and were analyzed via an unpaired *t*-test. The measurement data were analyzed using a chi square test in the form of frequency and percentage. *p* < 0.05 was considered to be statistically significant.

According to previous studies and results of pre-experiment, the mean cerebral oxygen saturation of control group was 70.2. It was predicted that the mean cerebral oxygen saturation of DEX group would been increased by 3.7. The variance of control group was 3.7 and the variance of DEX group was 4.1. The α of the bilateral tests was 0.05 and the test power 1-β was 0.9. The Two-Sample Assuming Unequal Variance Student’s t test was calculated via PASS15 and the sample size was 24 cases in each group. Considering loss to follow-up, the sample size was appropriately enlarged to 30 cases in each group.

## 3 Results

### 3.1 Analysis of the General Characteristics and the Perioperative Indexes

A total of 60 patients were recruited in the present study, with 30 patients allocated to the experimental group (DEX group) and 30 to the control group (GA group). There was no significant difference in the demographic analysis and the operation duration between the two groups. The anesthesia duration (*p* < 0.001) and the monitoring time in the anesthesia recovery room (*p* < 0.001) in the DEX group were significantly shorter than those in the GA group. The details are presented in Table 1.

**TABLE 1:** Analysis of the general characteristics and the perioperative indexes [*n* or (Mean ± SD)].

Index	Group DEX (*n* = 30)	Group GA (*n* = 30)	*p*
Age (y)	46.3 ± 10.4	43.7 ± 12.0	0.37
Body mass index (kg/m^2^)	25.0 ± 1.2	24.5 ± 1.8	0.21
Gender (M/F)	14/16	13/17	—
ASA grade (Ⅰ/Ⅱ)	9/21	8/22	—
Duration of anesthesia (min)	113.1 ± 17.4	127.9 ± 18.4	<0.001*
Duration of operation (min)	86.8 ± 11.3	86.8 ± 17.2	1
Monitoring time in the anesthesia recovery room (min)	23.2 ± 6.2	29.1 ± 6.4	<0.001*

Note:*p < 0.05 was statistically significant.

### 3.2 Analysis of Intraoperative Cerebral Oxygen Saturation and Hemodynamics

There was no significant difference in the cerebral oxygen saturation and the mean arterial pressure between the two groups at the initial stage of the anesthesia. As shown in Table 2, from the commencement of the anesthesia to the end of the operation, the cerebral oxygen saturation in the DEX was significantly higher than that in the GA group (*p* < 0.001), while the mean arterial pressure in the DEX group was higher than that in the GA group (*p* < 0.05).

**TABLE 2:** Analysis of intraoperative cerebral oxygen saturation and hemodynamics (Mean ± SD).

Time point	Cerebral oxygen saturation (%)		*p*	Mean arterial pressure (mmHg)		*p*
	Group DEX	Group GA		Group DEX	Group GA	
Before anesthesia	78.6 ± 1.2	77.9 ± 1.7	0.07	90.1 ± 5.8	93.0 ± 5.9	0.06
10 min after anesthesia	75.8 ± 1.7	76.1 ± 1.7	0.50	88.0 ± 5.9	83.0 ± 6.2	<0.01*
20 min after anesthesia	75.7 ± 1.6	72.0 ± 2.0	<0.001*	84.9 ± 6.1	79.1 ± 6.9	<0.01*
Before nerve block	75.9 ± 2.0	70.1 ± 2.3	<0.001*	83.6 ± 6.3	80.0 ± 7.1	0.04*
When cutting skin	76.2 ± 2.4	70.0 ± 2.9	<0.001*	85.9 ± 7.1	80.6 ± 7.0	0.01*
10 min after operation	76.0 ± 2.6	68.1 ± 2.8	<0.001*	84.4 ± 5.8	80.2 ± 7.2	0.02*
20 min after operation	74.9 ± 2.6	66.3 ± 4.5	<0.001*	83.3 ± 5.7	78.0 ± 6.3	<0.001*
30 min after operation	73.7 ± 3.1	65.8 ± 4.1	<0.001*	82.8 ± 6.4	77.7 ± 6.6	<0.001*
60 min after operation	72.5 ± 3.7	67.5 ± 4.2	<0.01*	83.4 ± 6.5	80.0 ± 6.5	0.05
90 min after operation	73.7 ± 3.2	69.0 ± 3.4	<0.001*	86.7 ± 6.2	81.2 ± 6.1	<0.001*
At the end of the operation	73.2 ± 4.3	70.0 ± 3.6	<0.001*	86.8 ± 6.3	81.6 ± 6.0	<0.001*

Note:*p < 0.05 was statistically significant.

### 3.3 Comparison of the Postoperative Visual Analogic Scale Scores

There was no significant difference in the VAS pain score at most time point after the operation between the two groups. However, The pain score of DEX group 12 h after operation significantly lower than that in the GA group (*p* < 0.05). The details are shown in Table 3.

**TABLE 3:** Comparison of the postoperative visual analogic scale scores (Mean ± SD).

Time point	Group DEX	Group GA	*p*
At the end of the operation (scores)	1.6 ± 1.1	1.7 ± 1.0	0.71
15 min after operation (scores)	1.9 ± 1.1	1.8 ± 1.1	0.73
30 min after operation (scores)	2.0 ± 1.0	1.9 ± 1.1	0.71
6 h after operation (scores)	2.3 ± 1.1	2.4 ± 1.2	0.74
12 h after operation (scores)	2.5 ± 0.8	3.1 ± 1.1	0.02*
24 h after operation (scores)	2.8 ± 1.6	3.3 ± 1.1	0.16
48 h after operation (scores)	2.5 ± 1.1	2.2 ± 1.6	0.40

Note:*p < 0.05 was statistically significant.

### 3.4 Analysis of the Complications

As illustrated in Table 4, compared with the DEX group, there were significant differences in the decrease in cerebral oxygen saturation and the mean arterial pressure in the GA group(*p* < 0.001), with a vasopressor used in 50% of the patients in this group to maintain the mean arterial pressure. In addition, the incidence of hypoxemia in the GA group was significantly higher than that in the DEX group (*p* < 0.05), and the incidence of the intervention of auxiliary respiration was higher in the former than in the latter (*p* < 0.05). Meanwhile, the incidence of postoperative nausea and vomiting in the DEX group was significantly lower than that in the GA (*p* < 0.01).

**TABLE 4:** Analysis of the complications [*n* (%)].

Index	Group DEX	Group GA	*p*
Decrease of cerebral oxygen saturation > 20%	1 (3.3)	17 (56.7)	<0.001*
Mean arterial pressure decreased > 20%	4 (13.3)	19 (63.3)	<0.001*
Vasopressor used	2 (6.7)	15 (50.0)	<0.01*
Apnea	2 (6.7)	4 (13.3)	0.70
Hypoxemia	3 (10.0)	10 (33.3)	0.02*
Auxiliary respiration	3 (10.0)	11 (36.7)	0.03*
Nausea and vomiting	10 (33.3)	21 (70.0)	0.01*

Note:*p < 0.05 was statistically significant.

## 4 Discussion

The application of general anesthesia in clinical procedures is undoubtedly common. According to previous research (Nam et al., 2011), the advantages of general anesthesia include that it is convenient to locate and adjust the patient’s position during the operation and that the implementation, maintenance, or termination of the anesthesia process can be adjusted according to the operation’s process and duration. However, in terms of intraoperative anesthesia management, general anesthesia has a great impact on the respiratory and circulatory systems. For one, the drugs used in general anesthesia can often cause clear fluctuations in blood pressure. Moreover, the patients will have no spontaneous breathing and must be assisted by the mechanical ventilation of the anesthesia machine, which poses a risk for patients with acute and chronic respiratory diseases and cardiovascular diseases and is prone to causing intraoperative hypotension and postoperative breathing inhibition or hypoxemia (Miskovic and Lumb, 2017; Wang et al., 2019). Furthermore, a number of studies have demonstrated that a general anesthesia involving the beach-chair position is prone to causing local cerebral ischemia and hypoxia, which may lead to temporary or permanent neurological complications (Janssen et al., 2014; Ersoy et al., 2016; Ding et al., 2017). All these conclusions were confirmed in the present study. Here, in the GA group, the incidence of a decrease in the cerebral oxygen saturation of >20% was 56.7%, while the incidence of a decrease in the mean arterial pressure of >20% was 63.3% and the incidence of hypoxemia was 33.3%.

Dexmedetomidine can inhibit the transmission of the action potential among neurons by activating the α 2-adrenoceptors in the spinal cord and the brain to achieve sedative and analgesic effects. In the present study, dexmedetomidine sedation combined with suprascapular nerve block and axillary block was found to have two main advantages.

First, the technique reduced the incidence of hyoxemia. Here, the dexmedetomidine provided an effective sedative effect on the basis of maintaining the spontaneous breathing, which not only shortened the anesthesia duration and postoperative monitoring time but also significantly reduced the incidence of hypoxemia. In addition, the area of interscalene brachial plexus block was in the nerve root and the radiation range not only included the shoulder and the surrounding tissues, but also included the sensory and motor areas of the forearm and hand. Given this wide blocking range, an improper operation could easily lead to injury of the nerve root. Moreover, the anatomical positions of the phrenic nerve and the interscalene brachial plexus are very close. When the brachial plexus of the interscalene is blocked, paralysis of the phrenic nerve can easily occur, and the respiratory movement will be weakened or may even stop. However, the shoulder joint and its surrounding tissues are mainly innervated by C5-C8 nerve roots and branch of brachial plexus, including suprascapular nerve and axillary nerve. The block area of the suprascapular nerve combined with the axillary nerve block only includes the sensory area of the shoulder and the surrounding tissue. The sensation and activity of the affected forearm and hand remained normal, which not only demonstrated an appropriate anesthetic and analgesic effect but also reduced the risk of the respiratory dysfunction caused by the paralysis of the phrenic nerve (Jiang et al., 2017).

Second, the application of dexmedetomidine will leave the patient in a conscious or a sedative state. Compared with when in a state of complete unconsciousness under general anesthesia, the patient’s neurohumoral regulation mechanism will be relatively complete and will have the capacity to maintain the stability of the hemodynamics within a certain range through self-regulation, not only ensuring the stability during the operation but also maintaining the cerebral blood perfusion (Ding et al., 2017). In the present study, at almost all time points following the commencement of the anesthesia, the patients in the DEX group had significantly higher cerebral oxygen saturation (*p* < 0.001) and mean arterial pressure. In fact, there were only four cases where there was a drop in the intraoperative mean arterial pressure to >20%, and only two patient was given a vasopressor. In contrast, in the GA group, while the end expiratory concentration of the sevoflurane was maintained as low as possible, the average arterial pressure was around 10–15 mmHg lower than that in the DEX group. Previous research has suggested that the decrease in the cerebral oxygen saturation is directly correlated with the mean arterial pressure (Koh et al., 2013). For every 10-mmHg decrease in mean arterial pressure, the cerebral oxygen saturation will decrease by around 0.1%, with the decrease potentially leading to an increase in the incidence of postoperative nausea, vomiting, and cognitive dysfunction. In the present study, the incidence of nausea and vomiting in the GA group was 70%, which was consistent with the findings in the existing literature.

However, while dexmedetomidine has a number of advantages in terms of sedation, various complications can emerge, including intraoperative apnea, which may be correlated with excessive sedation. In the present study, the apnea and the transient hypoxemia in the DEX group could be alleviated via tactile stimulation, the inhalation of high-flow oxygen, or respirator-assisted ventilation, without serious airway complications. However, the results suggested that while dexmedetomidine may maintain the patency of the airway as well as maintain spontaneous ventilation, apnea and hypoxemia may still occur when a high dose of dexmedetomidine is administered, which must be considered during clinical procedures.

## 5 Conclusion

In conclusion, the administration of dexmedetomidine combined with suprascapular nerve block and axillary nerve block during shoulder arthroscopy could reduce the incidence of postoperative respiratory complications. In addition, the approach demonstrated better hemodynamic stability, fully ensured the cerebral blood perfusion, and had better anesthetic and analgesic effects. However, it is crucial to remain vigilant in view of the potential emergence of apnea due to excessive sedation during the operation.

## Data Availability

The raw data supporting the conclusion of this article will be made available by the authors, without undue reservation.
